# The Mediating Role of Teacher Identification in the Relationship Between Psychological Distress and Teacher Satisfaction During COVID-19

**DOI:** 10.1177/00469580221110520

**Published:** 2022-07-29

**Authors:** Kyle Jackson, Zorina Noordien, Anita Padmanabhanunni, Tyrone B. Pretorius

**Affiliations:** 1 University of the Western Cape, South Africa

**Keywords:** professional identification, teaching satisfaction, mediation, hopelessness, depression

## Abstract

The current study examines the potential mediating role of professional identification of school teachers in the relationship between psychological distress and teaching satisfaction. Professional identification is the extent to which an individual identifies with a professional group, including the values, beliefs, and commitment the individual has in relation to the professional group. Professional identification has been linked to negative work outcomes, such as job performance and turnover intentions, as well as to adverse mental health outcomes. In the context of education, professional identification is the extent to which teachers identify with the teaching profession. Participants (N = 355) were school teachers in South Africa who completed the Center for Epidemiological Depression Scale, the Beck Hopelessness Scale, the Professional Identification Scale, and the Teaching Satisfaction Scale. Structural equation analysis showed that professional identification plays a fully mediational role in the relationship between psychological distress and teaching satisfaction. Interventions that strengthen teachers’ identification with the teaching profession should be considered a priority.

**What do we already know about this topic?**

Psychological well-being has been found to be highly correlated with job satisfaction. In this relationship, job satisfaction is often conceptualized as the independent variable that impacts psychological well-being. Various protective factors have been investigated to examine their potential mediating/moderating role in the relationship between job satisfaction and psychological well-being.

**How does your research contribute to the field?**

Firstly, we postulated that in the time of COVID-19 and the associated increase in psychological distress, that psychological well-being would be the independent variable affecting levels of job satisfaction in teachers. Secondly, we demonstrated that professional identification fully mediates the relationship between psychological distress and teaching satisfaction.

**What are your research’s implications toward theory, practice, or policy?**

The findings highlight the critical importance of professional identity, specifically teacher identity. In the context of a global devaluing of the teaching profession, this is an important finding that should lead to policy improvements to elevate the status of teachers in society. Interventions that strengthen teachers’ identification with the teaching profession should be considered a priority.

## Introduction

The global spread of the COVID-19 pandemic precipitated a range of public health responses aimed at curbing the spread of the virus. Governments around the world temporarily suspended face-to-face teaching to reduce transmission and new cases of infection.^1^ Since March 2020, approximately 90% of schools globally have been temporarily closed for some period of time, representing the most significant disruption of education in history.^2,3^

To ensure the continuity of the academic year, many schools transitioned to emergency remote online learning and teaching.^4^ For many teachers, this necessitated learning to use new technology, adapting lesson plans to an online format, and mastering a different pedagogical approach.^1^ Simultaneously, teachers were subjected to the same challenges experienced by the general population, including fears of contracting COVID-19, worries about the risk to their loved ones, uncertainty about the course of the pandemic, and managing work and domestic responsibilities (eg, childcare, home schooling, care for elderly loved ones).^5^ Emergency remote learning and teaching is resource-intensive and requires the commitment and involvement of students, parents, teachers, and school administrators. Emerging international research findings, for example^6^ indicate that virtual instruction may not have been as effective as initially assumed due to a range of factors, including teachers’ and families’ lack of means to access electronic technology and low levels of digital literacy.

The demands placed on teachers and the resources available to them have also varied during the pandemic. Recent studies have found that the multiple stressors experienced by teachers have led to increased adverse mental health outcomes among this group.^1,2^ In a meta-analysis, Ozamiz-Etxebarria^7^ reported that the prevalence of common mental health disorders (ie, depression and anxiety) was higher among teachers than among the general population during the initial waves of the COVID-19 pandemic. Other studies have confirmed that teachers have experienced increased stress, exhaustion, anxiety, depression, hopelessness, and sleep disturbance since the onset of the pandemic.^8^ Teachers with pre-existing mental health conditions were more adversely affected by the pandemic and resulting transition to emergency remote teaching than their peers.^9-11^ Some studies have suggested differences in the experience of primary versus secondary school teachers. Primary school teachers have been reported to experience increased levels of fear and anxiety owing to challenges with younger school-aged children’s ability to follow COVID-19 related safety protocols such as mask wearing and maintaining physical social distancing.^12^ Furthermore, primary school teaching requires high levels of creativity to engage younger children and work-from-home mandates and increased responsibility in the domestic sphere can affect teacher’s ability to effectively engage their creativity.^13^

The current study was conducted in South Africa, where school closures were first implemented in March 2020 as part of a strict national lockdown.^14^ The lockdown restrictions entailed travel prohibitions, mandated social distancing policies, and work-from-home directives. For the first time since the end of apartheid in the country in 1994, the army was deployed to support the police in enforcing the lockdown. These measures were intended to reduce viral transmission and thereby prevent an already overburdened public health care system from overwhelm. School closures were also based on the assumption of a mortality risk to teachers if conventional teaching were to continue.^5,15^ By August 2020, classroom-based teaching resumed on a limited basis (ie, groups of learners returned to school on alternative days or weeks). This shift was prompted by inequity in access to information technology and the reliance of many low-income families on school-based feeding programs.^16^ Data for the current study were collected during the third wave of the pandemic in South Africa (April–May 2021). Conventional face-to-face teaching had resumed, and preliminary reports suggested that teachers were experiencing heightened levels of anxiety and distress.^5^

The COVID-19 pandemic has drastically transformed and extended the scope of a teacher’s job, which may have implications for teachers’ professional identity and sense of satisfaction with their work.^5^ Professional identity entails the degree to which an individual identifies with their chosen profession and includes their appraisals about their job and commitment to their work.^17^ In this regard, therefore, the construct of professional identity differs from concepts such as organizational commitment or normative commitment. The former is conceptualized as an employee’s sense of personal attachment to the organization which includes embracing its goals and values while the latter refers to internalized sense of pressure to align one’s goals to the values and interests of the organization.^18^

Several studies^19-21^ have suggested that the migration to an online environment due to COVID-19 prevention measures has generated tension in teacher’s professional identities because they did not select to be online teachers. Instead, this role was thrust upon them and impacted on their ability to connect with their colleagues and students. Online classes were also found to limit teacher’s agency and capacity to enact their creativity and express themselves adequately toward their students.

Teaching satisfaction is defined as the extent of contentment derived from one’s profession and is related to congruence between an individual’s values and their occupation.^22^ Studies of professional identity and job satisfaction during the pandemic has mostly focused on frontline medical care workers, particularlynurses.^23,24^Available studies on teacher satisfaction have confirmed that levels of satisfaction dropped during the COVID-19 pandemic. For example, Hong et al,^25^ in a study of pre-school teachers, found that increased workload due to remote online teaching and learning negatively impacted on teacher satisfaction. In a systematic review of 662 articles on teacher satisfaction during the COVID-19 pandemic, Li and Yu^26^ concluded that teachers professional roles underwent profound changes and their workload increased substantially and this was associated with a decline in teacher satisfaction. Teachers have been called the “forgotten frontline workers” of the pandemic.^5,27^ Emerging research findings, for example, Fute et al^28^ suggest that teachers who highly valued their work and were engaged in their workplace experienced greater levels of job satisfaction and general wellbeing than their peers. These findings suggest that professional identity may be an important protective factor in teachers’ mental health outcomes and work satisfaction. The current study aims to extend existing research by examining the potential mediating role of teaching identification in the relationship between psychological distress and teaching satisfaction. The study is grounded in the theoretical framework provided by the Job Demands-Resources Model (JD-R).^29^ According to this framework, job demands are psycho-social characteristics of an organization or features of the work that require cognitive and emotional efforts and may lead to adverse psychological outcomes (eg, hopelessness and depression). On the other hand, job resources are aspects of the work that facilitate the achievement of work tasks, personal and professional growth and reduce the physical and psychological impact of job demands. Existing studies have confirmed the theoretical pathways proposed by the model. Job demand, for example, have been related to depression, substance abuse and psychological distress.^30^ In a similar vein, job resources (eg, perceived control over work tasks, supportive leadership, etc.) have been associated with increased motivation and organizational commitment. In the context of COVID-19 prevention measures, teachers face new work demands (eg, monitoring and implementing safety protocols among students) and have reduced autonomy due the changing course of the pandemic and the related responses of the government. It is therefore reasonable to conjecture that these new stressors may impact on their mental health. Job resources can buffer the adverse impact of job demands and can be found at an organization (eg, regular communication, provision of clear guidelines and personal protective equipment) or personal level.^29^ Professional identification and teaching satisfaction can be seen as job resources.

Based on the JD-R model and the findings of previous empirical studies, for example^12^ the current study aimed to investigate the role of job resources (ie, professional identification and teaching satisfaction) on psychological distress in the context of the current pandemic (job demands) and examined the following hypotheses:

H1: Hopelessness will be negatively related to teaching satisfaction.
H2: Depression will be negatively related to teaching satisfaction.
H3: Identifying with the teaching profession will be positively related to teaching satisfaction.
H4: When hopelessness is considered with professional identification, the negative relationship between hopelessness and teaching satisfaction will reduce or become nonsignificant, indicating a partial or full mediating role of professional identification in the hopelessness-teaching satisfaction relationship,
H5: When depression is considered with professional identification, the negative relationship between depression and teaching satisfaction will reduce or become nonsignificant, indicating a partial or full mediating role of professional identification in the depression-teaching satisfaction relationship.

**Figure 1.**
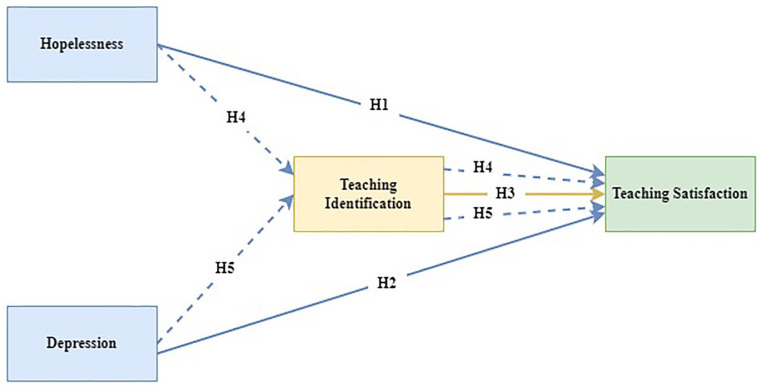
Conceptual model and hypotheses.

The conceptual model and the respective hypotheses above are reflected in Figure 1.

## Method

### Participants

The sample consisted of primary and secondary school teachers (N = 355) from across South Africa. The reported number of teachers in South Africans was 400 000 in 2021.^31^We targeted a sample size of 384 (5% margin of error and 95% confidence interval) but only secured a sample of 355. This represents a 5.09% margin of error with a 95% confidence interval. The majority of the sample resided in the Western Cape (82.3%) and was female (76.6%). The mean age of the sample was 41.89 (SD = 12.42, range: 23-73), and the mean number of years spent in the teaching profession was 15.7 (SD = 11.75, range: 1-48). The majority of respondents reported having lost a family member due to COVID-19 (63.9%), and 27% reported having lost a colleague.

### Instruments

In addition to a demographic survey, participants completed the Center for Epidemiological Depression Scale (CES-D),^32^ the Beck Hopelessness Scale (BHS),^33^ the Professional Identification Scale (PIS),^34^ and the Teaching Satisfaction Scale (TSS).^22^ The CES-D scale is a 20-item measure of depressive symptoms scored on a 4-point scale that ranges from *rarely or none of the time* (0) to *most or all of the time* (4).The CES-D was considered appropriate as it was developed for use in the general population and was also previously validated in South Africa.^35,36^ Example items of the CES-D are “I could not get going” and “I felt that everything I did was an effort.” In its original development study, the CES-D demonstrated satisfactory reliability (*α* = .85-.90), and its relationship with clinical ratings of depression provided evidence of validity.^32^ More recent studies have also reported satisfactory reliability indices of .88 and .81 for the scale.^37,38^ The CES-D has previously been used in South Africa and a Cronbach’s alpha of .90 was reported in that study.^39^

The BHS is a measure of hopelessness and general pessimism that consists of 20 items scored on a “true/false” dichotomous scale. The BHS was selected as the preferred measure of hopelessness as it is based on Beck’s cognitive theory of hopelessness and have previously been used in South Africa.^40,41^ Example items of the BHS include “Things just don’t work out the way I want them to” and “I just can’t get the breaks.” Beck et al^33^ reported an estimated internal consistency of .93 for the scale, and its validity has been demonstrated through correlations with clinical ratings of hopelessness and other measures of hopelessness. In more recent studies,^42,43^ similar satisfactory indices of reliability have been reported, namely .86 and .88. In South Africa, Padmanabhanunni and Pretorius reported an estimated internal consistency of *α* = .89 for the scale.^39^

The PIS is a measure of the extent to which participants identify with the teaching profession. It is based on the generic group identification scale developed by Brown et al^34^; in the TIS, the generic “group” is replaced with “teaching profession.” The PIS is theory driven as it is based on Social Identity Theory. It was thus considered as appropriate for the measurement of professional identity. The scale consists of 10 items measured on a 5-point Likert scale ranging from *never* (1) to *very often* (5). An example item of the TIS is “I am a person who feels strong ties to the teaching profession.” Brown et al^34^ reported a reliability coefficient (Cronbach’s alpha) of .71. Lu et al^44^ reported an alpha coefficient of .85 when using the scale with hospital nurses in China.

The TSS is a measure of participants’ satisfaction with their role as teachers. It consists of 5 items scored on a 5-point Likert scale ranging from *strongly disagree* (1) to *strongly agree* (2). Most measures of job satisfaction assess different domains in the work setting (eg, relationship with colleagues, work roles). The TSS, however is a brief, reliable and valid global assessment of job satisfaction.^22^ Example items of the TSS include “My conditions of being a teacher are excellent” and “So far, I have gotten the most important things I want as a teacher.” The original study reported a reliability coefficient of .77 and provided evidence of convergent and criterion-related validity.^22^ In more recent studies, reliabilities of .78 and .92 have been reported for the TSS.^45,46^

### Procedure

The current study was an internet-based, cross-sectional study. An electronic version of the instruments was developed and circulated to teachers through social media. In certain instances, teachers requested the instruments through email. The school liaison officer at the University of Western Cape also circulated the link to school teachers and administrators for whom she had contact information. Data collection occurred during April to June 2021 for a period of 3 months.

### Ethics

The Humanities and Social Sciences Ethics Committee of the University of Western Cape provided ethical approval to perform the study (ethics reference number: HS21/3/8). The participants were assured of the voluntary nature of participation and anonymity. They were required to provide informed consent on the first page of the survey before being allowed to proceed. As the questionnaires had the potential to cause distress, the participants were also provided with the contact details of counseling services that can be utilized free of charge.

### Data Analysis

Descriptive statistics, reliabilities (alpha and omega), and intercorrelations between study variables were determined using IBM SPSS Statistics for Windows (version 26; IBM Corp., Armonk, NY, USA). To examine the mediating role of professional identification in the relationship between psychological distress and teacher satisfaction, IBM SPSS Amos (version 26; IBM Corp.) was used. Bootstrapped confidence intervals and *P*-values were used to examine whether the indirect effects of depression and loneliness on teaching satisfaction (via professional identification) were significant. If the confidence intervals do not include zero, the indirect effects are considered significant.

## Results

The descriptive statistics, reliabilities, and intercorrelations for the study variables are reported in Table 1. The mean hopelessness score for the current study was 5.7 (SD = 4.9),which is significantly higher than scores previously reported^47-49^ for teacher samples, namely Ongen: ‾X = 4.48, SD = 4.27, *t* = 4.78, *P* < .001; Çavuş and Sarpkaya: ‾X = 3.96, SD = 4.12, *t* = 6.76, *P* < .001; Şengül et al: ‾X = 4.37, SD = 4.04, *t* = 5.20, *P* < .001. The following cutoff for the BHS is suggested in the literature: <3 = minimal hopelessness, 4 to 8 = mild hopelessness, 9 to 14 = moderate hopelessness, and ≥15 = severe hopelessness.^50^ Based on these proposed cutoff points, the current sample may be classified as follows: 42.5% minimal hopelessness, 31% mild hopelessness, 18.9% moderate hopelessness, and 7.6% severe hopelessness.

**Table 1.** Descriptive Statistics, Reliabilities, and Intercorrelations of Study Variables.

	1	2	3	4
1. Hopelessness	—			
2. Depression	.61^***^	—		
3. Professional identification	−0.39^***^	−0.41^***^	—	
4. Teaching satisfaction	−0.37^***^	−0.38^***^	.58^***^	—
Mean	5.7	21.9	40.1	17.3
SD	4.9	12.2	6.9	4.7
Alpha	.89	.92	.85	.87
Omega	.89	.93	.83	.87

*** *P* < .001.

The mean depression score was 21.9 (SD = 12.2). Compared to previous research, this score is similar to the mean score for a sample of teachers in an intervention study at baseline (Ebert et al: ‾X* =* 22.76, SD = 9.24, *t* = −1.23, *P* = ns),^37^ but significantly higher than those of other studies^51,52^ prior to the COVID-19 pandemic, for example Shen et al: ‾X = 18.48, SD = 9.4, *t* = 5.37, *P* < .001; Simor et al: ‾X = 15.45, SD = 9.56, *t* = 10.04, *P* < 001). The mean score in the current study is also significantly higher than that reported for a sample of teachers during COVID-19^53^: Truzoli et al: ‾‾X = 16.3, SD = 9.5, *t* = 8.73, *P* < 001). Regarding cutoff scores, Radloff initially indicated that ≥16 is a good indicator of being at risk for clinical depression.^32^ Based on this cutoff, 64.8% of the current study sample would be considered at risk. In a more recent study, Herniman et al suggested that ≥23 is an indicator of full-threshold comorbid depressive disorder.^54^ Based on this measure, 47.3% of the sample would be diagnosed with full-threshold comorbid depressive disorder.

The mean teaching satisfaction score was 17.3 (SD = 4.7). The scaled mean (‾X = 3.45) was significantly lower than the originally reported mean^22^: ‾X = 3.59, SD = 0.87, *t* = −2.80, *P* = .005). It was also significantly lower than those reported in other studies^46,55^ prior to the pandemic, namely Pervaiz et al: ‾X = 3.72, SD = 0.74, *t* = −5.43, *P* < .001; Han et al: ‾X = 4.11, SD = 0.71, *t* = −13.31, *P* < .001).

The reliabilities of the various scales (*α* = 0.85-0.92; *ω* = 0.83-0.93) can be considered highly satisfactory. They also compare favorably with previously reported reliabilities for the BHS,^50^ CES-D,^56^ TIS,^44^ and the TSS.^55^

The indices of psychological distress (ie, depression and hopelessness) were negatively related to teaching satisfaction (hopelessness: *r* = −.37, 95% CI [−0.46, −0.28], *P* < .001; depression: *r* = −.38, 95% CI [−0.47, −0.29], *P* < .001) and professional identification (hopelessness: *r* = −.39, 95% CI [−.48, −.30], *P* < .001; depression: *r* = −.41, 95% CI [−.50, −.32], *P* < .001) and positively related to each other (*r* = .61, 95% CI [0.54, 0.67], *P* < .001). Professional identification and teaching satisfaction were positively related to each other (*r* = .58, 95% CI [0.51, 0.65], *P* < .001). In other words, higher levels of depression and hopelessness were associated with lower levels of professional identification and teaching satisfaction, and higher levels of teacher identification were associated with higher levels of teaching satisfaction.

**Figure 2.**
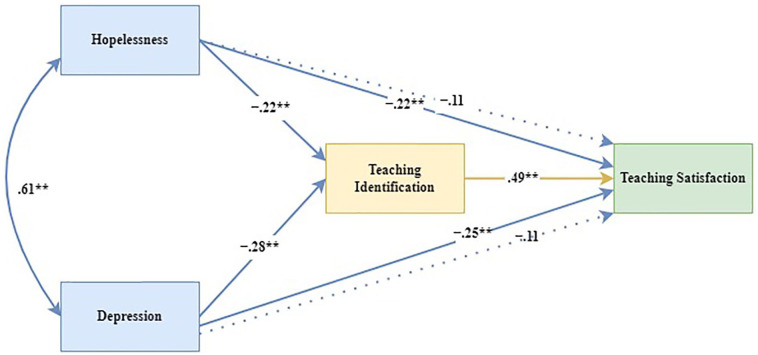
Structural equation model of the mediating role of teaching identification. *Note*. Solid line shows relationship between predictors and dependent variable in the absence of the mediator. Dotted line shows relationship between predictors and dependent variable in the presense of the mediator. Regression coefficient are standardized *P < .05, **P < .001.

A path analysis model was used to examine the mediating role of professional identification in the relationship between psychological distress and teacher satisfaction (see Figure 2). In this model, hopelessness and depression were operationalized as predictors, teaching satisfaction as the dependent variable, and professional identification as the presumed mediator. Based on Cooper et al’s suggestion that resources such as resilience and teaching identification should be viewed as causally antecedent to job stressors,^57,58^ we also examined a model in which teaching identification is the predictor, teaching satisfaction is the dependent variable, and the indices of psychological distress are the mediated pathways. However, in this model the standardized coefficients were non-significant (Professional Identification → Hopelessness → Teaching Satisfaction: *β* = .04, 95% CI [0.01, 0.06], *P* = .090; Professional Identification → Depression → Teaching Satisfaction: *β* = .05, 95% CI [0.01, 0.06], *P* = .052).

Figure 2 illustrates that the relationship between the predictors and the dependent variable was significant when considered in the absence of the mediator (hopelessness: *β* = −.22, 95% CI [−0.34, −0.10], *P* = .005; depression: *β* = −.25, 95% CI [−0.36, −0.13], *P* < .001). However, this association became nonsignificant in the presence of the mediator (hopelessness: *β* = −.11, 95% CI [−.22, .00], *P* = ns; depression: *β* = −.11, 95% CI [−0.21, −0.01], *P* = ns). This finding indicates that professional identification plays a fully mediational role in the relationship between the predictors and the dependent variable. The direct and indirect effects of hopelessness and depression, taking the mediator into account, are presented in Table 2.

**Table 2.** Direct and Indirect Effects of Predictors in the Presence of the Mediator.

Effect	Beta	SE	*β*	95% CI	*P*
Direct effects					
Hopelessness → Professional identification	−.309	0.089	−.220	[−0.322, −0.114]	.004
Hopelessness → Teacher satisfaction	−0.107	0.063	−.113	[−0.223, −0.004]	.091
Depression → Professional identification	−.159	0.036	−.281	[−0.375, −0.172]	.001
Depression → Teaching satisfaction	−.042	0.023	−.110	[−0.213, −0.013]	.060
Professional identification → Teaching satisfaction	.331	0.033	.492	[0.404, 0.563]	.001
Indirect effects					
Hopelessness → Professional identification → Teaching satisfaction	−.102	0.030	−.108	[−0.155, −0.057]	.003
Depression → Professional identification → Teaching satisfaction	−.053	0.014	−.138	[−0.076, −0.031]	.001

Table 2 confirms the nonsignificant association between indices of psychological distress and teaching satisfaction in the presence of the mediator, as indicated in Figure 2. It also shows significant indirect effectsof hopelessness (*β* = −.11, 95% CI [−.16, −.06], *P* = .003) and depression (*β* = −.14, 95% CI [−.08, −.03], *P* < .001) on teaching satisfaction. This finding indicates that professional identification fully mediates the impact of indices of psychological distress on teaching satisfaction; in other words, indices of psychological distress impact teaching satisfaction through the mediator of professional identification. These findings support all the postulated hypotheses.

H1: Hopelessness was negatively related to teaching satisfaction (*β* = −.22, 95% CI [−.34, −.10], *P* = .005).
H2: Depression was negatively related to teaching satisfaction (*β* = −.25, 95% CI [−.36, −.13], *P* = .005).
H3: Identifying with the teaching profession was positively related to teaching satisfaction (*β* = .49, 95% CI [0.40, 0.56], *P* = .001).
H4: Professional identification fully mediated the relationship between hopelessness and teaching satisfaction (*β* = −.11, 95% CI [−.16, *−*.06], *P* = .003).
H5: Professional identification fully mediated the relationship between depression and teaching satisfaction (*β* = −.14, 95% CI [−.08, −.03], *P* = .001).

## Discussion

The current study examined the potential mediating role of identification with the teaching profession in the relationship between psychological distress and teaching satisfaction. The results supported all the proposed hypotheses. First, the study confirmed that psychological distress had a significant impact on teaching satisfaction. Although this relationship is not unique to the COVID-19 context of learning and teaching, it is potentially more salient because the educational landscape was profoundly impacted by pandemic-related prevention measures. This could negatively impact on levels of teaching satisfaction and enhance psychological distress. It is likely that the return to conventional classroom-based schooling may have led teachers to experience increased fear about exposure to the virus and concern about their ability to protect themselves and their loved ones.^8,59^ These fears may have been compounded by contextual factors such as insufficient access to personal protective equipment and under-resourced school environments characterized by poor infrastructure, limited access to running water, and insufficient space to adhere to social distancing protocols.^8^ These conditions, coupled with the South African government’s pressure on teachers to “save the academic year,” may have heightened distress and adversely impacted job satisfaction among teachers.^60^ According to cognitive social theory, individuals’ appraisals of negative life events influence their psychological well-being.^61^ It is therefore probable that teachers who appraised themselves as having little agency and limited capacity to engage in self-protective behaviors may have experienced heightened levels of distress in the form of hopelessness and depression.

Second, the study findings indicate that teaching identification significantly mediated the impact of psychological distress on teaching satisfaction. This finding suggests that teaching identification may serve as a protective factor for mental health outcomes. Professional identity entails a complex synthesis of thoughts, experiences, and behaviors associated with a particular profession and work role.^62^ High levels of professional identity are associated with a clear understanding of the job, as well as flexibility to adapt and adjust role expectations.^63^ Teachers who strongly identify with their profession are more likely than their peers to experience a sense of belonging and congruence between their perceptions of their self-identity and professional identity.^59,62^ Low levels of professional identity are associated with feelings of insecurity regarding one’s professional value, which can lead to a state of indifference and consequent hopelessness and depression.^62,63^

Within the context of COVID-19 and the rapid shift from face-to-face teaching to remote learning modalities, professional identity is fundamental to the context of teaching. In consideration of the significant historical socioeconomic disparities in South Africa, Moodley^64^ reported on the creative and innovative solutions of some South African teachers. These solutions included the use of WhatsApp to engage with learners and parents, consultations with colleagues around the world to explore options for remote teaching, and provision of learning materials using WhatsApp as a medium. Teachers with high professional identities tend to appraise their job and the stressors thereof as an opportunity to positively impact the lives of their learners.^5,65^ Such teachers are more likely than others to adjust their role expectations and adapt to the challenges of the job, particularly within the context of the COVID-19 pandemic.^63^ Teachers who have a strong sense of professional identity may perceive remote learning as an opportunity to protect lives by curbing the spread of the virus.^5^ These teachers may have also appraised the reopening of schools as necessary to promote the educational attainment of students who do not have access to resources to engage with remote learning.^5,66^

Professional identification plays an important role in influencing mental health outcomes and job satisfaction for teachers in South Africa. Identifying the beliefs and values that underpin teaching identification in this context and encouraging teachers to strengthen these internal capacities could enhance adaptation and coping. Cognitive-behavioral and mindfulness-based interventions have demonstrated strong efficacy in building resilience resources. These modalities proved effective in South Africa in pre-COVID-19 research.^67^ Future research is needed to assess the utility of this approach in promoting teacher well-being in the current global context.

### Limitations

The study has several limitations. A cross-sectional survey design was used, which limits the extent to which causal inferences can be made. Longitudinal studies are needed to confirm these findings. The study was also conducted in a single geographical area. It would be beneficial for future studies to recruit a more geographically diverse participant sample.

### Conclusion

The study findings suggest that teacher identification is a potential protective factor for mental health outcomes. Future studies should explore the nuances and causal factors of depression and hopelessness among teachers, as well as the intervention strategies associated with these mental health outcomes.
